# Short-Term Mortality in Hospitalized Patients with Congestive Heart Failure: Markers of Thrombo-Inflammation Are Independent Risk Factors and Only Weakly Associated with Renal Insufficiency and Co-Morbidity Burden

**DOI:** 10.3390/jcdd11030093

**Published:** 2024-03-20

**Authors:** Jose Iglesias, Nelson Okoh, Song Peng Ang, Cristina A. Rodriguez, Jia Ee Chia, Jerrold S. Levine

**Affiliations:** 1 Department of Medicine Rutgers Health Community Medical Center Internal Medicine Residency Program, Community Medical Center RWJBH, Toms River, NJ 08757, USA; nelsonokoh07@gmail.com (N.O.); songpengang@gmail.com (S.P.A.); cristinarod94@gmail.com (C.A.R.); 2 Department of Medicine, Hackensack Meridian School of Medicine, Nutley, NJ 07110, USA; 3 Department of Medicine, Texas Tech University Health Science Center, El Paso, TX 79905, USA; jchia@ttuhsc.edu; 4 Department of Medicine, Division of Nephrology, University of Illinois Chicago, Chicago, IL 60612, USA; jslevine@uic.edu; 5 Department of Medicine, Division of Nephrology, Jesse Brown Veterans Affairs Medical Center, Chicago, IL 60612, USA

**Keywords:** congestive heart failure, biomarkers, platelet/lymphocyte ratio, thrombin time, D-dimer, 28-day mortality, inflammation

## Abstract

Congestive heart failure (CHF) is associated with significant morbidity and mortality. There has been renewed interest in using thrombo-inflammatory markers as prognostic tools in patients with CHF. To determine if thrombo-inflammatory markers are independent risk factors for 28-day mortality in hospitalized CHF patients, we retrospectively analyzed admission data extracted from 2008 consecutive patients admitted with a diagnosis of CHF to Zigong Fourth People’s Hospital. Multivariate Cox proportional hazards analysis demonstrated that the thrombo-inflammatory markers thrombin time, platelet/lymphocyte ratio (PLR), and D-dimer level were independent predictors of mortality. In addition, variables reflecting the severity of CHF (New York Heart Association class > 2), impaired renal function (elevated serum creatinine [SCr]), impaired organ perfusion (elevated BUN), and chronic liver disease were also independent predictors of mortality. Thrombo-inflammatory biomarkers were only weakly associated with SCr and the burden of co-morbidity, suggesting that thrombo-inflammation may in large part be attributable to CHF itself and that, moreover, its presence may confer an increased risk of mortality. Further large-scale prospective studies are needed to determine the existence and the consequences of a thrombo-inflammatory phenotype among patients with CHF.

## 1. Introduction

Congestive heart failure (CHF) remains a global healthcare problem with a prevalence of about 1–2% of the population. Recent studies show that approximately 38 million people worldwide (almost twice the population of the state of New York) have this disease, with about 6.2 million living in the USA [1,2,3]. An estimated 21 out of 1000 older adults in the USA and 15 to 20 out of 1000 in Europe have CHF [3,4,5,6,7,8]. CHF remains a leading cause of hospitalization, averaging about 1 million hospitalizations per year in the USA, with death rates varying from 10% after one year to close to 50% after five years of diagnosis [1,3,4,5,7,9].

As a global burden, the prevalence of CHF continues to increase, largely due to aging populations. The prevalence of CHF is expected to reach as high as 10% among patients over 70 years [1,2,3,4,5,7,10]. Despite advances in treatment approaches and guidelines, mortality rates of CHF remain remarkably high.

Several large registries have characterized the epidemiology, clinical profile, and outcomes for patients with CHF [2,11,12,13,14,15]. Overall, independent risk factors for mortality include hemodynamic aberrations, impaired ejection fraction, markers of renal dysfunction, elevated blood urea nitrogen (BUN), hyponatremia, hyperaldosteronism, comorbidities such as diabetes and hypertension, rheumatic valvular heart disease, smoking, coronary artery disease, atrial fibrillation, and central sleep apnea [3,13,16,17,18,19,20,21,22,23,24].

Recently, there has been renewed interest and data supporting the use of thrombo-inflammatory markers obtained from routine complete blood counts, such as the neutrophil/lymphocyte ratio and the platelet/lymphocyte ratio, as well as other thrombo-inflammatory biomarkers, including C-reactive protein (CRP) and D-dimer, as prognostic tools in CHF patients [25,26,27,28,29,30,31]. It remains unclear whether the inflammatory state is an inherent aspect of CHF, an independent predictor of outcome, and/or a reflection of the burden of comorbidities. Similar uncertainty surrounds the possible existence of a prothrombotic state in CHF. Thus, a thrombo-inflammatory state may exist in CHF patients and impact clinical outcomes. With this in mind, we evaluated admission laboratory studies, epidemiologic data, and comorbidities obtained on admission from 2008 consecutive patients admitted with CHF to Zigong Fourth People’s Hospital from December 2016 to June 2019 [32].

## 2. Methods

### 2.1. Data Source

To determine if thrombo-inflammatory markers are independent risk factors for mortality in CHF patients, we obtained data on admission from a de-identified database extracted from the electronic health records of 2008 consecutive patients admitted with a diagnosis of CHF to Zigong Fourth People’s Hospital from December 2016 to June 2019 [32]. These data were made available by the original investigators and creators of the database [32]. This study and the provision of its database to other investigators was approved by the ethics committee of Zigong Fourth People’s Hospital (Approval Number: 2020-010) [32]. Informed consent was waived due to the retrospective design of this study. This study complies with the Declaration of Helsinki.

### 2.2. Definition of CHF

CHF was defined according to criteria put forth by the European Society of Cardiology. Briefly, any signs or symptoms of CHF, such as dyspnea, edema, hepatojugular reflux, orthopnea, exercise intolerance, or an S3 gallop, that were corroborated by objective evidence of CHF, such as echocardiography or stress echo, abnormal chest X-ray, and elevated brain natriuretic peptide (BNP) biomarkers, were considered diagnostic of CHF [32].

### 2.3. Data Selection

Data analyzed from the dataset obtained during index admission fell into several broad categories: demographic data, baseline clinical data extracted from the medical record on the day of hospital admission, comorbidities, laboratory findings, and outcomes. Clinical data obtained on the day of hospital admission included pulse, respiratory rate, systolic and diastolic blood pressure, mean arterial blood pressure, body mass index (BMI), type of heart failure, and New York Heart Association (NYHA) cardiac function. Comorbidities were extracted from the electronic medical records and included a history of myocardial infarction (MI), peripheral vascular disease (PVD), cerebrovascular disease, chronic obstructive pulmonary disease (COPD), diabetes, moderate-to-severe chronic kidney disease (CKD, stage 3 or higher), and chronic liver disease. For patients already carrying a diagnosis of CHF, CHF was considered a co-morbidity. Patients who had no history of CHF prior to admission were documented as having new-onset CHF. As the original database did not contain a diagnosis of coronary artery disease (CAD), we created a CAD equivalent, defined as a history of MI, PVD, and/or cerebrovascular disease. Hypertension was defined as a systolic blood pressure ≥ 140 mm Hg on admission.

Laboratory data analyzed from the first day of hospital admission included serum creatinine (SCr), blood urea nitrogen (BUN), cystatin-C, uric acid, estimated glomerular filtration rate (eGFR), white blood cell count (WBC), hemoglobin, hematocrit, platelet count, neutrophil count, lymphocyte count, D-dimer level, thrombin time, fibrinogen, high-sensitivity troponin, calcium, potassium, sodium, brain natriuretic peptide (BNP), high-sensitivity C-reactive protein (CRP), and albumin. As age impacts D-dimer levels, we also created an age-adjusted D-dimer level. Age-adjusted analysis of the impact of D-dimer was based on the fact that the normal range for D-dimer increases with age, with the upper limit of normal increasing by 100 ng/mL for each decade after the age of 50. We therefore adjusted for age range by multiplying the patient’s measured D-dimer level by the ratio of the upper limit of normal for ages ≤ 50 divided by the upper limit of normal for the patient’s decade of age (e.g., a 59-year-old patient’s D-dimer level would be multiplied by 500/600) [33]. eGFR was calculated employing the modified diet in renal disease equation (MDRD) for Chinese individuals (per personal communication with database creator Z. Zhang [32]). Hematologic ratios, which have been shown to be markers of inflammation, such as the neutrophil-lymphocyte ratio (NLR) and the platelet-lymphocyte ratio (PLR), were calculated.

### 2.4. Statistical Analysis

Summary statistics were computed for 28-day survival and non-survival groups. We performed both univariate and multivariate analyses. We employed the Shapiro–Wilks test to assess if the data were normally distributed. As no continuous variables were normally distributed, continuous variables were all expressed as medians with interquartile ranges and were compared by the Mann–Whitney-U test. Categorical values were compared with Pearson’s chi-squared or Fisher’s exact test. Pearson’s bivariate correlation test was used to determine the degree of correlation between the Charlson co-morbidity index (a predictor of 10-year survival in patients with multiple comorbidities) and various thrombo-inflammatory biomarkers [34].

Variables determined to be significant by univariate analysis at a p-value less than 0.05 were candidates for multivariate analysis. Multivariate Cox proportional hazards with forward variable selection was performed to identify variables independently predictive of survival at 28 days. For continuous variables, the hazard ratio (HR) represents the relative amount by which the probability of obtaining the outcome variable increases or decreases when the independent variable is changed by exactly one unit. HRs and their 95% confidence intervals (CIs) were determined through exponentiation of the regression coefficient and its upper and lower CIs, respectively. Inspection of the Schoenfeld residuals plot found the proportional hazards assumptions to be met. The sensitivity and specificity of continuous variables independently associated with survival were reported, and their receiver operating characteristic (ROC) curves as a function of threshold were analyzed. The sensitivity and specificity of binary variables were calculated as the number of True positives/True positives + False negatives and the number of True negatives/True Negatives + False positives, respectively. Missing values in the case of standard laboratory results were not imputed and accounted for less than 3% of all measurements. Missing data for several non-standard laboratory tests (e.g., CRP and D-dimer) were found to be missing completely at random (MCAR) according to Little’s MCAR test [35]. Missing values of tests who were missing by greater than 3% were substituted using multiple imputations and are reported in Supplementary Table S1. All statistical analyses were performed with IBM SPSS version 28 software (IBM SPSS Inc., Chicago, IL, USA).

## 3. Results

### 3.1. Univariate Analysis of Factors Associated with 28-Day Mortality

We first evaluated the impact of clinical and laboratory features from the day of admission (Table 1), as well as patient demographics and co-morbidities (Table 2), on 28-day mortality. Features significantly associated with 28-day mortality according to the univariate analysis included the following: increased uric acid, increased cystatin C, increased WBC count, increased platelet count, increased neutrophil count, increased thrombin time, increased fibrinogen, increased high-sensitivity troponin, decreased calcium, increased potassium, decreased sodium, increased BNP, increased CRP, decreased albumin, increased SCr, increased BUN, increased D-dimer, and increased PLR (Table 1). It is pertinent to note that after adjusting D-dimer for age, the D-dimer differences in survivors and non-survivors remained statistically significant. Comorbidities associated with 28-day mortality included increased NYHA classification, moderate-to-severe CKD, chronic liver disease, and acute kidney injury (AKI) (Table 2).

### 3.2. Bivariate Relationship between Charlson Co-Morbidity Index, eGFR, and Thrombo-Inflammatory Biomarkers

To determine the degree to which increased levels of thrombo-inflammatory bio-markers reflect co-morbidity burden and/or CKD, we performed a bivariate analysis assessing the correlation between thrombo-inflammatory biomarkers and the Charlson co-morbidity index (Table 3) and eGFR (Table 4). Although a statistically significant association existed between the Charlson co-morbidity index and neutrophil count, fibrinogen level, NLR, CRP, and PLR, the coefficient of correlation (R) was relatively small, ranging from 0.07 to 0.1 (Table 3). Similar results were found for the association between eGFR and lymphocyte count, neutrophil count, D-dimer, NLR, CRP, and PLR, for which the R values ranged from 0.07 to 0.14 (Table 4). Therefore, the elevation of these biomarkers cannot be solely explained by the burden of comorbidities and/or presence of CKD.

### 3.3. Multivariate Analysis of Factors Independently Associated with 28-Day Mortality

**Figure 1:**
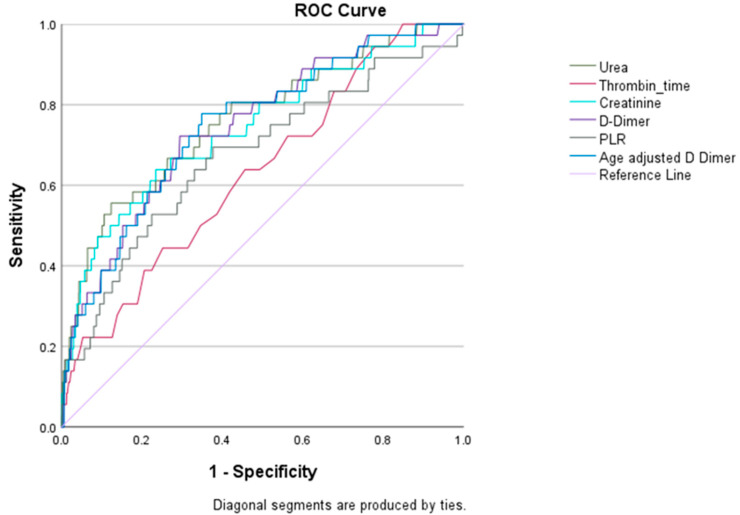
Receiver operating characteristic (ROC) curves for variables independently associated with 28-day mortality. Color code: urea—dark green, thrombin time—red, creatinine—light blue, D-dimer—purple, platelet/lymphocyte ratio (PLR)—dark grey, age-adjusted D-dimer—dark blue.

In order to determine which of the univariate factors associated with 28-day mortality were independent predictors of mortality, we performed a Cox proportional hazards analysis with stepwise forward regression. The independent predictors of 28-day mortality, in descending order of hazards ratio, were NYHA class > 2, the presence of chronic liver disease, elevated SCr, elevated thrombin time, elevated BUN, elevated PLR, and elevated D-dimer (Table 5). The limited sensitivity and specificity analysis of variables independently associated with 28-day mortality may have been impacted by the small number of events (Table 6, Figure 1). Separate ROC curves displayed individually for each variable are provided in Supplementary File S1.

## 4. Discussion

We determined the risk factors for 28-day mortality among 2008 consecutive patients admitted to a single hospital and diagnosed with CHF. The database we used in our retrospective analysis was unique in its inclusion of thrombo-inflammatory bio-markers, which are typically not included in most cohorts of CHF patients. In accordance with previous studies, we found that the independent predictors of short-term mortality included variables reflective of the severity of CHF (NYHA > 2), impaired renal function (elevated SCr), impaired organ perfusion (elevated BUN), and liver disease. In addition, several biomarkers indicative of a thrombo-inflammatory state were also independent predictors of mortality, namely, elevations in thrombin time, PLR, and D-dimer levels. While a thrombo-inflammatory state has been associated with both CKD and the burden of co-morbidities [27,28,36,37,38,39], the association between each of the thrombo-inflammatory biomarkers included in this study and CKD or the Charlson co-morbidity index was very weak (R < 0.15), suggesting that thrombo-inflammation is itself a major predictor of short-term mortality and that the co-morbidity burden and/or the presence of CKD cannot alone explain the elevation in these biomarkers.

Over the last several decades, experimental and clinical data have demonstrated that CHF is accompanied by a thrombo-inflammatory state. Biomarkers associated with an inflammatory state, such as TNF-α, IL-6, and IL-1β, are of prognostic importance in CHF patients [40,41,42]. Similarly, Cugno et al. have shown that patients with CHF exhibit elevated markers of coagulation, fibrinolysis, and endothelial activation, as well as increased levels of pro-inflammatory cytokines [43]. The mechanisms by which CHF predisposes patients to thrombo-inflammation are diverse and involve a complex interplay between hemodynamic and cardiac factors, such as increased wall tension, mechanical stress, volume overload, activation of the renin–angiotensin–aldosterone system (RAAS), and mitochondrial oxidative stress, with eventual organ cross-talk and activation of the innate immune system, especially the NLRP3 inflammasome [42,44,45,46,47,48]. In addition, comorbidities commonly present in patients with CHF, such as diabetes mellitus, CKD, hypertension, obesity, liver disease, and COPD, are themselves associated with increased inflammation [39]. Hematologic manifestations of inflammatory pathway activation include lymphopenia and elevations of neutrophils and platelets [25,26,49,50,51,52,53], the latter two cells being important mediators of inflammation through their release of pro-inflammatory cytokines [54,55,56]. Lindeman et al. and others have demonstrated that platelets produce and release IL 1β under the right conditions [57,58]. Moreover, the activity and functional interrelationships among platelets, neutrophils, and lymphocytes have been associated with clinical outcomes in patients with cardiovascular disease [54,55,56]. The ratios between these three cell populations, such as the NLR and PLR, both easily obtained through routine blood work, may serve as surrogate biomarkers of an inflammatory state [25,26,49,50,52,53,55,59,60].

Although our study identified PLR as an independent predictor of 28-day mortality, other studies evaluating the role of PLR as a surrogate marker of inflammation predictive of CHF outcomes have yielded conflicting results [26,51,61,62,63]. A study by Durmus et al. found that an elevated PLR in CHF patients was an independent predictor of short-term, but not long-term, mortality [64]. However, Heidarpour et al. and Pourafkari et al. found that PLR predicted neither short-term nor long-term outcomes in CHF patients [61,62]. Delcea et al., in a comprehensive review evaluating over 17,000 patients, showed not only that PLR was associated with short-term mortality outcomes [65], but also that during long-term follow-up, ranging from 6 months to 5 years, PLR was an independent predictor of long-term mortality [65]. Demir et al. revealed that an increase in PLR in patients with acute CHF was associated with an increase in hospital and total mortality [66]. Finally, Ye and Huang and others found that an increase in PLR was an independent predictor of poor clinical outcomes and mortality [43,53,63].

The presence of both SCr and BUN as independent predictors merits comment. Several studies have revealed that BUN and creatinine levels have distinct roles in predicting outcomes [27,28]. These studies suggest that BUN, in addition to being a marker of overall kidney function, might also be a good indicator of the kidney’s response to impaired organ perfusion and neuro-humoral activation in patients with CHF [27,28,67,68]. Ren et al., in a study of over 600 patients admitted with worsening CHF, observed that BUN was positively associated with all-cause mortality, with the risk of death increasing by 1.6% for every 1 mmol/L increase in BUN concentration [43]. The study also found that BUN levels were similar to BNP in predicting all-cause mortality [67]. Fonarow et al., in an analysis of a validation cohort from the ADHERE registry, containing over 38,000 patients admitted with worsening CHF, observed that out of 39 variables analyzed, the best predictor of mortality was high admission levels of BUN [13].

Of interest, our results showed that the presence of chronic liver disease was also independently associated with 28-day mortality. The coexistence of liver disease and cardiac failure is not uncommon, given the complex, bi-directional interaction between the heart and liver and the broad number of diseases, both acute and chronic, that affect these two organs [69]. Two significant forms of liver dysfunction observed in cardiac patients are congestive hepatopathy and acute cardiogenic liver injury/hypoxic liver injury [69,70]. The incidence of congestive hepatopathy in patients with CHF ranges from 15 to 60% [69,70,71]. Its pathophysiology likely involves the retrograde transmission of elevated central venous pressure into the hepatic veins and venules [70].

### Study Strength and Limitations

The major strength of our current study lies in our ability to evaluate the role of thrombo-inflammatory variables, such as D-dimer, thrombosis time, CRP, and fibrinogen, which are not typically available in earlier cohorts of CHF patients. This study’s major limitation is that the data were extracted retrospectively from a single hospital center within a relatively homogeneous population. While this means that our results may not be extrapolatable to other populations, the homogeneity of our study may also have reduced genetic heterogeneity as a confounding variable and, therefore, made it easier to uncover a role for thrombo-inflammation in mortality from CHF. While the database provided information on medications prescribed on the day of admission, it did not distinguish whether they were started in hospital or continued from home medications. In addition, the database lacks information as to whether medications were continued or discontinued during hospitalization. Thus, we are uncertain what medications were prescribed on discharge. Given this lack of information, we felt that the inclusion of medications, even on the day of admission, was fraught with too much uncertainty and potential error to be included as a predictor in our model. We employed eGFR as a measure of renal function in our analysis. However, eGFR has its limitations, as it may over- or under-estimate renal function in various settings. Unfortunately, due to patient privacy concerns, we did not have access to individual patients’ age information for analysis. Consequently, we were unable to assess age as a continuous variable, thereby limiting our ability to determine its importance when incorporated into the Cox proportional hazards analysis. Also, since our study’s data were extracted from electronic medical records, not all patient data were complete, particularly laboratory studies that are not routinely ordered for every patient. We attempted to replace missing data using multiple imputations, with the sole purpose of this approach being hypothesis generation.

It is important to note that we adjusted the level of D-dimer based on decade of age rather than individual age, since D-dimer increases with age. In 2010, Duoma et al. highlighted the effectiveness of an age-adjusted D-dimer cut-off for predicting pulmonary embolism [33]. Similarly, adjusting the cut-off value for D-dimer measurement in older populations could enhance its clinical utility as a predictor of mortality in persons with CHF.

## 5. Conclusions

In summary, the current study supports the hypothesis that CHF is associated with a thrombo-inflammatory phenotype/state, manifesting as elevated PLR, thrombin time, and D-dimer levels, and that this thrombo-inflammatory state is an independent risk factor for 28-day mortality among patients hospitalized for CHF. In accord with earlier studies, we also identified increased severity of CHF, the presence of CKD, hepatic dysfunction, and elevated BUN (a putative marker of organ under-perfusion) as additional independent risk factors for 28-day mortality. As the biomarkers of a thrombo-inflammatory state were only weakly associated with SCr and the Charlson co-morbidity index, it appears that thrombo-inflammation may not only be attributable in large part to CHF itself, but may also confer an increased risk of mortality. The current study suggests that simple, inexpensive laboratory tests indicative of thrombo-inflammation and the assessment of comorbidities may identify patients at increased risk of early mortality. Because the small number of events in our study may have affected the determination of sensitivity and specificity, further large-scale, prospective studies with a greater number of events are needed to determine the existence, clinical utility, and consequences of identifying a thrombo-inflammatory phenotype among patients with CHF.

## Figures and Tables

**Table 1:** Correlation of clinical and laboratory values obtained on the day of admission with mortality at 28 days.

	Survivors (N = 1971)	Non-Survivors (N = 37)	*p*-Value
**Clinical variables**			
PULSE (beat/min)	82 (70, 98)	88 (70, 102)	0.28
SBP (mm Hg)	130 (114, 147)	126 (100, 141)	0.15
DBP (mm Hg)	76 (66, 85)	72 (60,84)	0.13
MAP	93 (83, 104)	90 (75, 99)	0.1
BMI	21 (18.5, 23)	21 (18, 23)	0.7
CHARLSON INDEX	2 (1, 2)	2 (1, 3)	0.1
**Laboratory variables**			
URIC ACID (mg/dL)	7.7 (6.0, 9.6)	10 (7.0, 13)	0.00001
eGFR (mL/min)	65 (42, 90)	34 (16–65)	0.00001
CYSTATIN C (mg/L)	1.5 (1.2, 2.2)	2.4 (1.6, 4.3)	0.00001
WBC COUNT (10^9^/L)	6.5 (5, 8.6)	9.2 (6.3, 11.3)	0.001
HEMATOCRIT (%)	36 (32, 40)	35 (24–40)	0.2
LYMPHOCYTE COUNT (10^9^/L)	0.9 (0.6, 1.3)	0.83 (0.4, 1.2)	0.1
HEMOGLOBIN (g/dL)	11.7 (10, 13)	11 (7.8, 13.3)	0.12
PLATELET COUNT (10^9^/L)	134 (101, 176)	178 (114, 239)	0.005
NEUTROPHIL COUNT (10^9^/L)	4.9 (3.6, 6.7)	7.3 (5, 10)	0.00001
THROMBIN TIME	17 (16, 18)	18 (17, 18)	0.003
FIBRINOGEN (mg/dL)	300 (250, 370)	360 (270, 450)	0.04
HIGH-SENSITIVITY TROPONIN (pg/mL)	0.05 (0.023, 0.12)	0.13 (0.08, 0.23)	0.00001
CALCIUM (mg/dL)	9.2 (8.8, 9.6)	8.8 (8.4, 9.2)	0.003
POTASSIUM (mEq/L)	3.9 (3.5, 4.3)	4.5 (3.8, 5.3)	0.00001
SODIUM (mEq/L)	139 (136, 141)	136 (133, 139)	0.001
BNP (pg/mL)	744 (300, 1726)	1448 (741, 3387)	0.001
CRP (mg/L)	9.2 (3.9, 29)	43 (8, 77)	0.004
ALBUMIN (g/dL)	3.7 (3.3, 4.0)	3.4 (3.2, 3.7)	0.004
CREATININE (mg/dL)	0.98 (0.73, 1.4)	1.8 (1.0, 3.2)	0.00001
BUN (mg/dL)	48 (35, 68)	94 (55, 139)	0.00001
D-DIMER (ng/mL)	1200 (790, 2130)	3055 (1385, 10,142)	0.00001
AGE-ADJUSTED D-DIMER (ng/mL)	753 (500, 1355)	1853 (1045, 5698)	0.00001
NEUTROPHIL/LYMPHOCYTE RATIO (NLR)	5.1 (3.2, 8.5)	8.3 (5.6–19.4)	0.57
PLATELET/LYMPHOCYTE RATIO (PLR)	145 (96, 223)	235 (132, 379)	0.0001

Abbreviations: BMI = body mass index, BNP = brain natriuretic peptide, BUN = blood urea nitrogen, CRP = C-reactive protein, DBP = diastolic blood pressure, eGFR = estimated glomerular filtration rate, MAP = mean arterial pressure, SBP = systolic blood pressure.

**Table 2:** Correlation of demographic features and co-morbidities with mortality at 28 days.

	Survivors (N = 1971)	Non-Survivors (N = 37)	*p*-Value	Odds Ratio	95% CI
AGE ≥ 69	1434 (73%)	28 (76%)	0.69	1.16	0.55–2.5
GENDER (M)	824 (42%)	21 (57%)	0.07	1.83	0.95–3.52
NYHA > 2	1619 (82%)	36 (%)	0.014	7.82	1.07–57
BIVENTRICULAR HEART FAILURE	1453 (74%)	27 (73%)	0.57		
LEFT HEART FAILURE	467 (24%)	10 (27%)	0.57		
RIGHT HEART FAILURE	51 (2.6%)	0	0.57		
HYPERTENSION (SBP ≥ 140 mm Hg)	743 (38)	13 (35%)	0.75	0.89	0.45–1.8
ACUTE MI	140 (7.1%)	3 (8%)	0.74	1.15	0.35–3.8
NEW DIAGNOSIS OF CHF	132 (7%)	4 (11%)	0.32	1.66	0.6–4.6
PVD	99 (5%)	2 (5%)	0.71	1.1	0.26–4.5
CEREBROVASCULAR DISEASE	145 (7.4%)	5 (13.5%)	0.19	1.97	0.75–5.13
COPD	231 (12%)	2 (5.4%)	0.31	0.43	0.10–1.80
CAD (Surrogate)	715 (36%)	15 (40%)	0.59	1.2	0.61–2.3
DIABETES	458 (23%)	8 (22%)	1	0.91	0.41–2.0
MODERATE-TO-SEVERE CKD	455 (23%)	19 (51%)	0.00001	3.5	1.8–6.7
CHRONIC LIVER DISEASE	78 (4%)	6 (16%)	0.004	4.7	1.9–11.6
ACUTE RENAL FAILURE	5 (0.3%)	2 (5.4)	0.007	22.5	4.2–120

Abbreviations: CAD = coronary artery disease, CHF = congestive heart failure, CKD = chronic kidney disease, COPD = chronic obstructive pulmonary disease, M = male, MI = myocardial infarction, NYHA = New York Heart Association, PVD = peripheral vascular disease.

**Table 3:** Correlation between thrombo-inflammatory biomarkers and the burden of comorbidities (Charlson co-morbidity index).

	Pearson Correlation Coefficient	*p*
Charlson co-morbidity index	1	
Lymphocyte count	−0.040	0.078
Platelet count	−0.010	0.66
Neutrophil count	0.106	0.001
Thrombin time	0.036	0.173
Fibrinogen	0.072	0.001
D-dimer	0.036	0.127
NLR	0.10	0.00001
CRP	0.10	0.00001
PLR	0.072	0.001

Abbreviations: CRP = C-reactive protein, NLR = neutrophil/lymphocyte ratio, PLR = platelet/lymphocyte ratio.

**Table 4:** Correlation between thrombo-inflammatory biomarkers and eGFR.

	Pearson Correlation Coefficient	*p*
eGFR	1	
Lymphocyte count	0.12	0.00001
Platelet count	0.04	0.10
Neutrophil count	−0.10	0.00001
Thrombin time	−0.01	0.60
Fibrinogen	−0.04	0.07
D-dimer	−0.12	0.00001
NLR	0.14	0.00001
CRP	−0.07	0.001
PLR	−0.07	0.00001

Abbreviations: CRP = C-reactive protein, eGFR = estimated glomerular filtration rate, NLR = neutrophil/lymphocyte ratio, PLR = platelet/lymphocyte ratio.

**Table 5:** Factors independently predictive of 28-day mortality as determined by Cox proportional hazards analysis.

	Beta	SE	Sig.	HR	95% CI	
					Lower	Upper
Thrombin time (s)	0.024	0.006	0.0001	1.02	1.012	1.038
Creatinine (mg/dL)	0.277	0.112	0.0131	1.32	1.060	1.642
D-dimer (ng/mL) *	0.000	0.000	0.0012	1.00	1.000	1.000
Urea (mg/dL)	0.012	0.004	0.0018	1.01	1.004	1.020
Platelet/lymphocyte ratio	0.003	0.001	0.0001	1.003	1.001	1.004
Liver disease	1.654	0.471	0.0004	5.23	2.078	13.166
NYHA > 2	2.413	1.057	0.0224	11.17	1.408	88.579

* D-dimer, HR = 1.00004, 95% CI = 1.0000–1.0001 for every 1 ng/mL change. Abbreviations: CI = confidence interval, HR = hazards ratio, CI = confidence interval. (All variables significant according to univariate analysis in Table 1 and Table 2 were entered into the Cox proportional hazards analysis).

**Table 6:** Sensitivity and specificity of variables independently associated with 28-day mortality.

Variables	Sensitivity	Specificity
Urea	0.56	0.87
Thrombin time	0.46	0.75
Creatinine	0.68	0.72
D-dimer	0.72	0.71
Platelet/lymphocyte ratio	0.68	0.62
Age-adjusted D-dimer	0.72	0.66
NYHA class > 2	0.97	0.18
Liver disease	0.16	0.97

## Data Availability

No new data was obtained in the current study. The data for the current study are available from PhysioNet^¶^ (https://physionet.org, accessed on 27 January 2024) upon request after filling out the data use agreement. Goldberger, A., Amaral, L., Glass, L., Hausdorff, J., Ivanov, P. C., Mark, R., … & Stanley, H. E. (2000). PhysioBank, PhysioToolkit, and PhysioNet: Components of a new research resource for complex physiologic signals. Circulation [Online]. 101 (23), pp. e215–e220.

## Supplementary Materials

The following supporting information can be downloaded at: https://www.mdpi.com/article/10.3390/jcdd11030093/s1, Figure S1: ROC curve for Urea demonstrating an area under the curve (AUC) of 0.76; Figure S2: ROC curve for Thrombin time demonstrating an AUC of 0.647; Figure S3: ROC curve for creatinine demonstrating an AUC of 0.74; Figure S4: ROC curve for D-Dimer demonstrating an AUC 0.75; Figure S5: ROC curve for PLR demonstrating an AUC 0.67; Figure S6: ROC curve for age adjusted D-Dimer demonstrating an AUC 0.75; Table S1: Missing values in our data set.
