# Supplementary material for: Short-Term Mortality in Hospitalized Patients with Congestive Heart Failure: Markers of Thrombo-Inflammation Are Independent Risk Factors and Only Weakly Associated with Renal Insufficiency and Co-Morbidity Burden
Source: J Cardiovasc Dev Dis. 2024 Mar 20;11(3):93. doi: 10.3390/jcdd11030093 (PMC10971481; doi:10.3390/jcdd11030093)
Supplement: Supplementary file 1 [file jcdd-11-00093-s001.zip › jcdd-2869446-supplementary.pdf]

Supplemental File S-1

Supplemental Fig. S-1) Receiver operating characteristics curve (ROC) for urea

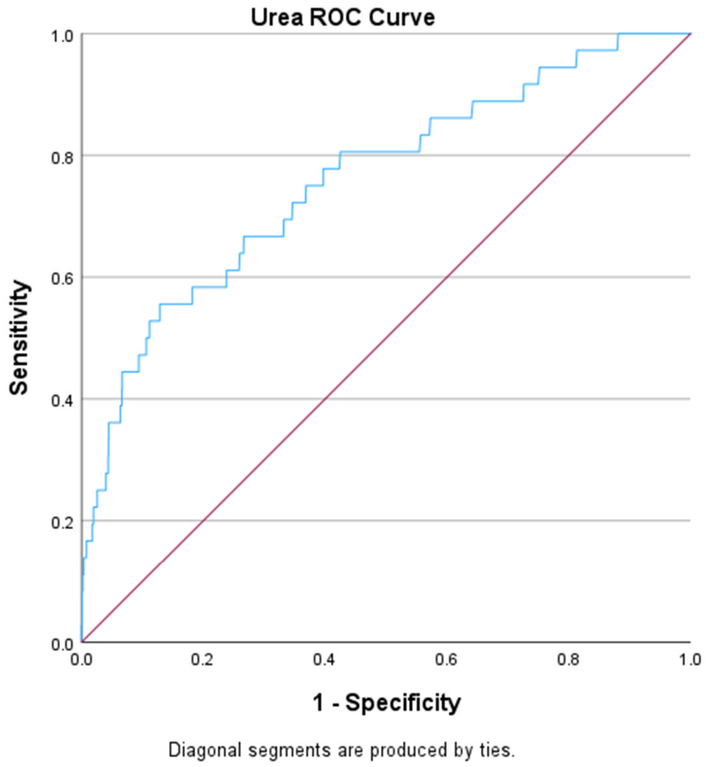

Supplemental Fig. S-1) ROC curve for Urea demonstrating an area under the curve (AUC) of 0.76

Supplemental Fig. S-2) Receiver operating characteristics curve (ROC) for Thrombin Time

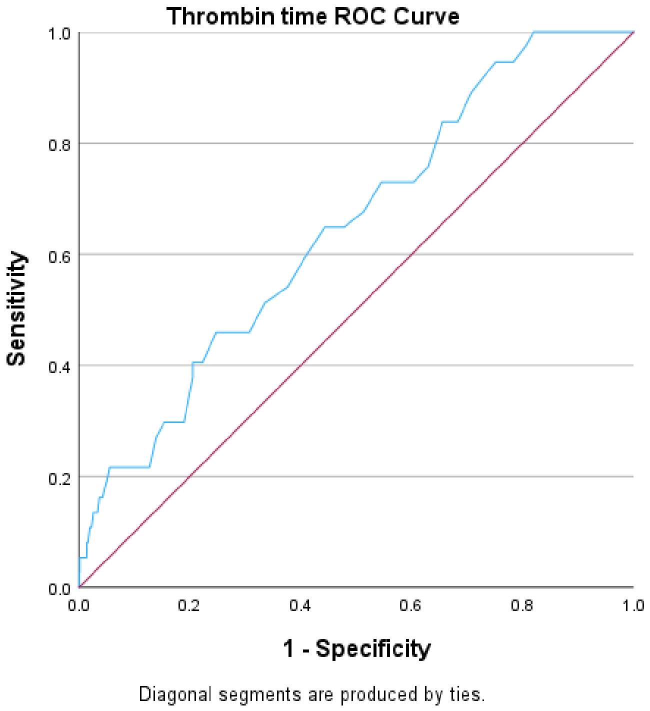

Supplemental Fig. S-2) ROC curve for Thrombin time demonstrating an AUC of 0.647

Supplemental Fig. S-3) Receiver operating characteristics (ROC) curve for Creatinine

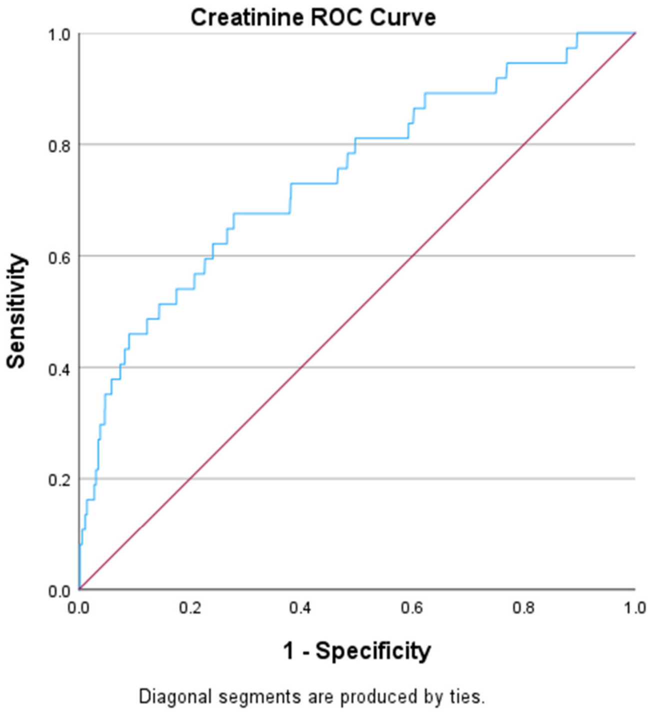

Supplemental Fig. S-3) ROC curve for creatinine demonstrating an AUC of 0.74

Supplemental Fig. S-4) Receiver operating characteristic curve (ROC) for D-Dimer

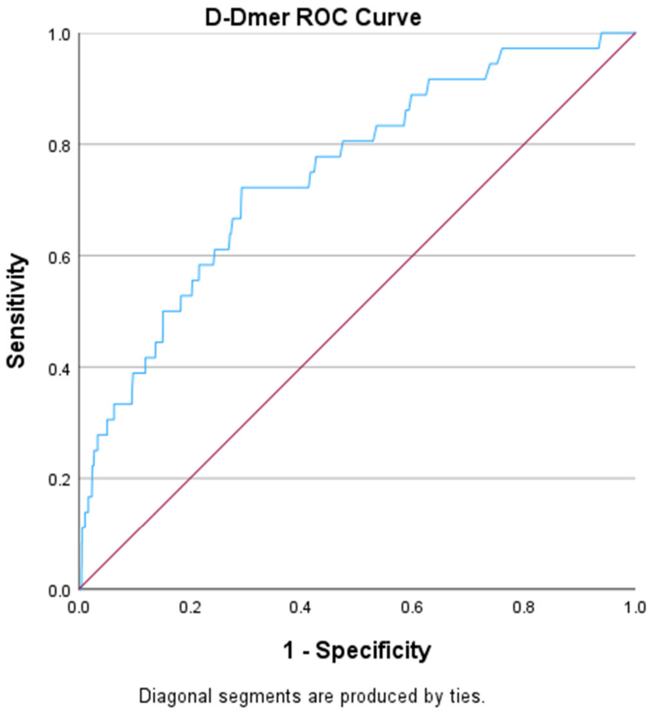

Supplemental Fig. S-4) ROC curve for D-Dimer demonstrating an AUC 0.75

Supplemental Fig S-5) Receiver operating characteristic curve for PLR

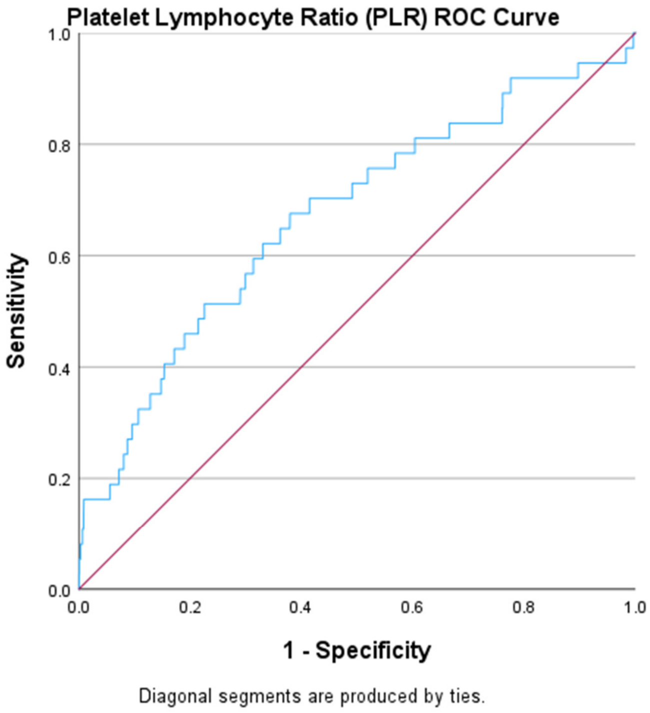

Fig. S-5) ROC curve for PLR demonstrating an AUC 0.67

Supplemental Fig. S-6) Receiver operating characteristic curve for Age adjusted D-Dimer

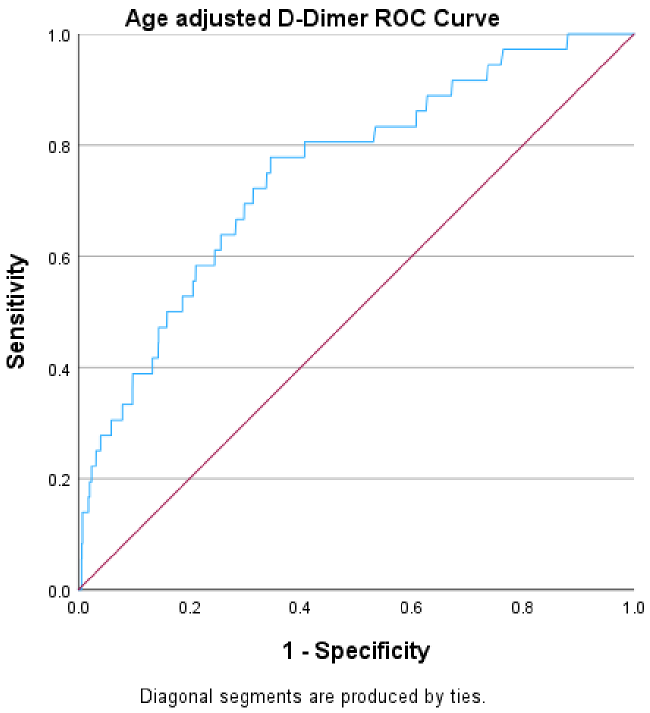

Supplemental Fig. S-6) ROC curve for age adjusted D-Dimer demonstrating an AUC 0.75

Table S-1 . Missing values in our data set.

		Missing	
	N	Count	Percent
D_dimer	1840	168	8.4
Thrombin_time	1974	34	1.7
Fibrinogen	1974	34	1.7
Troponin I	1929	79	3.9
BNP	1973	35	1.7
CRP	941	1067	53.1

Urea	1985	23	1.1
Uric_acid	1985	23	1.1
GFR	1945	63	3.1
Cystatin-C	1967	41	2.0
WBC	1981	27	1.3
Lymphocyte	1981	27	1.3
Hemoglobin	1980	28	1.4
Platelet	1981	27	1.3
Neutrophil_count	1981	27	1.3
Potassium	1997	11	.5
Sodium	1997	11	.5
Albumin	1906	102	5.1
Total_bilirubin	1906	102	5.1
Creatinine	1985	23	1.1
NLR	1981	27	1.3
PLR	1981	27	1.3

NLR Neutrophil lymphocyte ratio

PLR Platelet lymphocyte ratio
